# Deviations in Hippocampal Subregion in Older Adults With Cognitive Frailty

**DOI:** 10.3389/fnagi.2020.615852

**Published:** 2021-01-13

**Authors:** Mingyue Wan, Yu Ye, Huiying Lin, Ying Xu, Shengxiang Liang, Rui Xia, Jianquan He, Pingting Qiu, Chengwu Huang, Jing Tao, Lidian Chen, Guohua Zheng

**Affiliations:** ^1^College of Nursing and Health Management, Shanghai University of Medicine and Health Sciences, Shanghai, China; ^2^College of Rehabilitation Medicine, Fujian University of Traditional Chinese Medicine, Fuzhou, China

**Keywords:** cognitive frailty, hippocampal subregion, volume, diffusion tensor imaging, correlation

## Abstract

**Background:**

Cognitive frailty is a particular state of cognitive vulnerability toward dementia with neuropathological hallmarks. The hippocampus is a complex, heterogeneous structure closely relates to the cognitive impairment in elderly which is composed of 12 subregions. Atrophy of these subregions has been implicated in a variety of neurodegenerative diseases. The aim of this study was to explore the changes in hippocampal subregions in older adults with cognitive frailty and the relationship between subregions and cognitive impairment as well as physical frailty.

**Methods:**

Twenty-six older adults with cognitive frailty and 26 matched healthy controls were included in this study. Cognitive function was evaluated by the Montreal Cognitive Assessment (MoCA) scale (Fuzhou version) and Wechsler Memory Scale-Revised Chinese version (WMS-RC), while physical frailty was tested with the Chinese version of the Edmonton Frailty Scale (EFS) and grip strength. The volume of the hippocampal subregions was measured with structural brain magnetic resonance imaging. Partial correlation analysis was carried out between the volumes of hippocampal subregions and MoCA scores, Wechsler’s Memory Quotient and physical frailty indexes.

**Results:**

A significant volume decrease was found in six hippocampal subregions, including the bilateral presubiculum, the left parasubiculum, molecular layer of the hippocampus proper (molecular layer of the HP), and hippocampal amygdala transition area (HATA), and the right cornu ammonis subfield 1 (CA1) area, in older adults with cognitive frailty, while the proportion of brain parenchyma and total number of white matter fibers were lower than those in the healthy controls. Positive correlations were found between Wechsler’s Memory Quotient and the size of the left molecular layer of the HP and HATA and the right presubiculum. The sizes of the left presubiculum, molecular of the layer HP, and HATA and right CA1 and presubiculum were found to be positively correlated with MoCA score. The sizes of the left parasubiculum, molecular layer of the HP and HATA were found to be negatively correlated with the physical frailty index.

**Conclusion:**

Significant volume decrease occurs in hippocampal subregions of older adults with cognitive frailty, and these changes are correlated with cognitive impairment and physical frailty. Therefore, the atrophy of hippocampal subregions could participate in the pathological progression of cognitive frailty.

## Introduction

Cognitive frailty (CF) is a major subtype of frailty. According to the International Academy on Nutrition and Aging (I.A.N.A.) and the International Association of Gerontology and Geriatrics (I.A.G.G.), in 2013, cognitive frailty was first defined as a clinical syndrome characterized by physical frailty and cognitive impairment among older adults, excluding Alzheimer’s disease and other dementias (Kelaiditi et al., 2013). Epidemiological surveys estimated the prevalence rate of cognitive frailty to be 3–9.8% in the general older adult population, whereas the figure was much higher, 10.7–40%, in the clinical setting (Ma et al., 2017; Malek et al., 2019). Cognitive frailty can accelerate cognitive impairment and physical frailty in older adults, is associated with a decline in activities of daily living and quality of life, and increases the risk of dementia, falls, disability, and death (Roppolo et al., 2017). As human life expectancy continues to increase, the prevalence of cognitive frailty is rapidly increasing and has become one of the biggest health threats in the 21st century (Bishop et al., 2010). However, cognitive frailty (encompassing both the physical and the cognitive domain) seems a useful objective in terms of prevention considering the reversibility. Previous study has shown that there are some useful clinical, biological, and imaging markers of cognitive frailty (Ruan et al., 2017). Therefore, it is important for early intervention to find an effective brain imaging biomarker that can enable early identification of older adults with cognitive frailty.

Previous studies found that the hippocampus is the core brain area related to cognition, but it is not a unified brain region; rather it is composed of several subregions with specific histological features, and distinct pathways affect its overall function (Foo et al., 2017; Lisman et al., 2017). Therefore, the function of the hippocampus depends on its own internal structures, such as hippocampal subfields, and the connections of its surrounding structures with other parts of the brain (Foster et al., 2019). For example, a recent work showed that the anterior hippocampus contributed to global memory, perception, imagination and recall of scenes and events (Zeidman and Maguire, 2016). The posterior hippocampus was found to support fine and perceptual detailed memory (Sekeres et al., 2018). The head of the hippocampus is related to logical memory, while the body and tail of the hippocampus are related to visual memory (Travis et al., 2014). The CA1 of the hippocampus is connected to the posterior cingulate cortex, which can regulate episodic memory (Yamawaki et al., 2019). Therefore, the distinct hippocampal subregions are related to different types of cognition. However, its mechanism in cognitive frailty remains unclear.

Previous research has shown that physical decline in the process of aging may be at least partly due to damage to the brain or nerve function, not just disorders of skeletal muscle, and the hippocampus might be involved in the regulation of human body functions (Bland and Oddie, 2001; Clark et al., 2019). It is well known that the hippocampus is devoted to balance regulation and sensory motor integration (Bland and Oddie, 2001), while the hippocampus body and anterior cingulate gyrus are involved in memory and executive function (Mcgough et al., 2018). Moreover, a reduced integrity of the gray matter in these two regions was positively correlated with greater stride variability in elderly adults, which indicates the role of cognitive function in motor control (Rosso et al., 2014). A positive correlation between the left hippocampus volume, especially of the left CA1, CA2 and subiculum, among elderly adults and the balance composite score was also observed (Rehfeld et al., 2017). Older adult with a strong sense of fatigue have smaller hippocampal volume than control older adults who did not have a strong sense of fatigue (Carvalho et al., 2017). Therefore, the hippocampus or its subregions might contribute to the process of both cognitive and physical decline. We speculated that the hippocampus or its subregions play an important role in the pathogenesis of cognitive frailty. To address this hypothesis, we performed high-resolution structural MRI scans in a group of older adults with cognitive frailty as well as in controls. We used volumetry analysis to assess different aspects of the hippocampus and hippocampal subregions. The above indicators as well as behavioral indicators were subjected to correlation analysis to clarify the relationship. The whole brain index evaluation included analysis of cortical thickness, the number of white matter fibers in the whole brain and the proportion of brain parenchyma.

## Materials and Methods

### Participants

This cross-sectional study recruited 26 older adults with cognitive frailty and 26 matched healthy controls between April 2019 and September 2019 from communities in Fuzhou City, Fujian Province, China. All 52 participants included in this study participated in complete assessment scale and neuroimaging data collection. This study was approved by the ethics committee of The Second People’s Hospital Affiliated with Fujian University of Traditional Chinese Medicine. Written informed consent was obtained from all participants before participation.

All cognitive frailty participants met the following inclusion criteria (Won et al., 2018): Chinese version of EFS score ≥5 points; Fuzhou version of the MoCA score ≤26 scores; Clinical Dementia Rating (CDR) Scale score = 0.5 (i.e., just mild cognitive impairment) and age ≥60 years. The inclusion criteria for the age- and education-matched controls were an EFS score <5; a Fuzhou version of the MoCA score >26; and a CDR Scale score = 0.

Individuals were excluded when they met one of the following conditions: history of mental illness (such as personality disorder, schizophrenia, etc.); serious depression (Beck Depression Inventory scale score >10); mild dementia and above (CDR Scale score >0.5); history of alcohol or drug abuse; use of drugs that influence cognitive function; serious organ failure, cerebral hemorrhage, sequelae of cerebral infarction; unsuitability for MRI scanning (such as fixed metal dentures, pacemakers, etc.); and participation in another clinical trial.

### Cognitive and Physical Frailty Assessment

Global cognitive ability and memory were evaluated by using the Fuzhou version of the MoCA (Fang et al., 2017) and Wechsler’s Memory Scale (Elwood, 1991). Global cognitive function was measured using the Fuzhou version of the MoCA scale, which is a brief test that evaluates visuospatial/executive functions, naming, verbal memory registration and learning, attention, abstraction, delayed verbal memory and orientation. MoCA scores range from 0 to 30, and a higher score indicates better cognitive function; scores lower than 26 points are considered to represent mild cognitive impairment (Nasreddine et al., 2005). Memory was evaluated using the WMS-RC, which can assess the long-term, short-term, and immediate memory. Wechsler’s Memory Quotient ranges from 51 to 150, with higher scores indicating better memory. Physical frailty was assessed through the Chinese version of the EFS (Rolfson et al., 2006) and the grip strength test. The frailty index was assessed using the Chinese version of the EFS. The EFS had 11 items including 9 domains (cognitive ability, general health status, functional independence, social support, drug use, nutrition, emotions, control and functional performance), the scores range from 0 to 17 and a higher score indicates higher level of frailty. Grip force was measured using an electronic grip force meter (CAMRY-EH101). In the test, the participants were asked to use the maximum strength three times on each side, and the mean value was taken.

### MRI Data Acquisition

All participants underwent T1 and DTI imaging on a Siemens Prisma 3.0 T magnetic resonance scanner (Siemens Medical System, Erlangen, Germany) at Fujian Province Rehabilitation Hospital. The parameters of T1 imaging were as follows: repetition time (TR) = 2,300 ms, echo time (TE) = 2.27 ms, flip angle = 8°, slice thickness = 1.0 mm, field of view (FOV) = 250 × 250 mm, matrix = 256 × 256, voxel size = 0.98 × 0.98 × 1 mm^3^, and number of slices = 160. For DTI, the parameters were as follows: TR = 8,000 ms, TE = 64 ms, FOV = 224 × 224 mm, slice thickness = 2.0 mm, gap = 0 mm, slice number = 75, and slice order = interleaved.

### Imaging Processing

**FIGURE 1:**
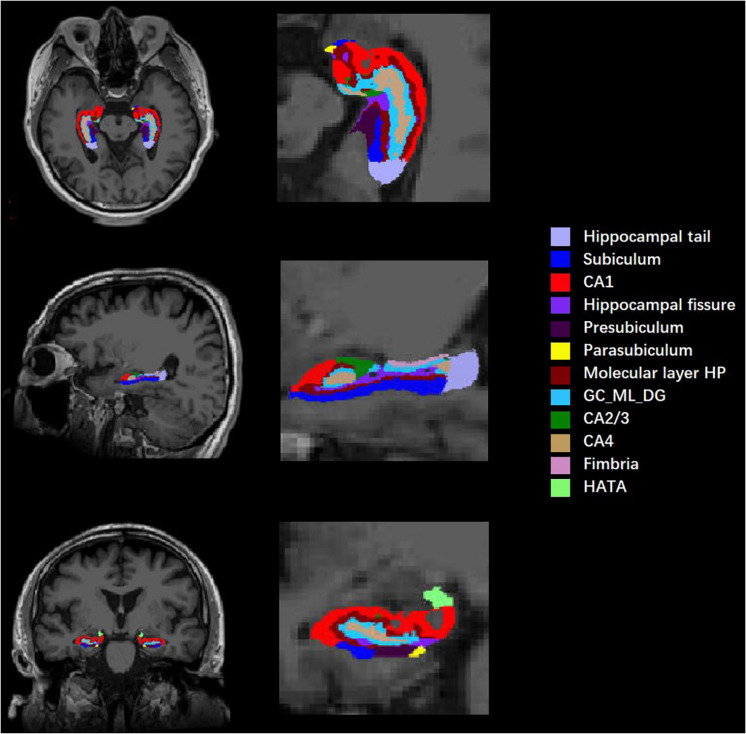
Diagram of hippocampal segmentation. T1 images of hippocampal subregions from view angles of sagittal, coronal, and axial planes and their magnifications.

All T1-weighted images were processed by publicly available FreeSurfer software (Version 6.0.0)^1^ using the default settings. Before data preprocessing, image format conversion and image quality assessment were needed. Then, the command “recon-all” in FreeSurfer 6.0.0 was used for volumetric segmentation, specifically including Talairach transformation, intensity normalization, skull stripping, volumetric registration, segmentation of gray and white matter and separation of the boundary, automatic subcortical segmentation, topology adjustment to fill and cut, and finally smoothing. Subcortical structures were segmented with a non-linear warping atlas. Subsequently, a probabilistic atlas and a modified version of Van Leemput’s algorithm were applied to segment the hippocampus (Iglesias et al., 2015; Saygin et al., 2017) into 12 subfields in each hemisphere: hippocampal tail, subiculum, CA1, hippocampal fissure, presubiculum, parasubiculum, molecular layer of the HP, granule cell layer and molecular layer of the dentate gyrus (GC-ML-DG), CA2/3, CA4, fimbria, and HATA (shown in Figure 1). CA2 and CA3 were combined due to a lack of clear contrast, and the alveus volume was removed on account of the thin shape and unreliable segmentation. To reduce the effect of individual differences, the total intracranial volume (TIV), including the brain parenchyma and cerebrospinal fluid (CSF), was estimated as a covariate.

Cortical thickness analysis was performed using the Computational Anatomy Toolbox (CAT12)^2^. CAT12 is based on the free and open source Statistical Parametric Mapping (SPM12)^3^, which was run in MATLAB 2016^4^. The gray matter, white matter and CSF were segmented automatically and then applied to the MNI template space with non-linear deformation and affine registration. CAT12 estimated cortical thickness based on the projection-based thickness (PBT) method (Dahnke et al., 2013), which includes partial volume correction. The gray matter and white matter were regarded as the brain parenchyma, and the ratio of brain parenchyma volume to TIV was referred to as the proportion of brain parenchyma, which was used to measure the degree of brain atrophy.

DTI images were analyzed by Fslutils (FSL)^5^, with processing including data format conversion, data quality inspection, eddy current correction, brain extraction, diffusion index estimation, diffusion tensor reconstruction, and whole brain fiber track reconstruction. Then, the total number of brain fibers for each participant was obtained.

### Statistical Analysis

SPSS 21.0 (IBM Corp., Armonk, NY, United States) was used for data analysis, and a *p* < 0.05 was considered significant. Quantitative data are expressed as the mean ± standard deviation and analyzed by independent samples *t*-test or the Mann-Whitney *U*-test. Categorical variables are described as frequencies and were compared using the chi-square test. The statistical threshold was adjusted by Bonferroni correction, and a *p* < 0.05/12 was taken as statistically significant when analyzing the volume of hippocampal subregions.

To explore the relationship between hippocampal subregion volume and cognition and physical frailty state, we conducted a partial correlation analysis between them with age, gender, years of education, Beck Depression Scale and TIV as covariates.

## Results

### Demographic Characteristics and Performance Variables

The MoCA scores and grip strength in the cognitive frailty group were significantly lower than those in the healthy control group (*t* = 11.98, *p* < 0.001; *t* = −2.380, *p* = 0.021). The score of the MQ scores in the cognitive frailty group were significantly lower than those in the healthy control group (*t* = −9.767, *p* < 0.001). The FI score of the cognitive frailty group was significantly higher than that of the healthy control group (*t* = 8.263, *p* < 0.001). There was no significant difference in gender, age, years of education or Beck Depression Scale score between the two groups. Regarding certain aspects of brain macroscopic indicators, the proportion of brain parenchyma and total number of white matter fibers in the cognitive frailty group were significantly lower than those in the healthy control group (*t* = −3.273, *p* = 0.002; *t* = −8.835, *p* < 0.001), while the total intracranial volume and cortical thickness were not significantly different between the two groups (see Table 1).

**TABLE 1:** Demographic and performance variables between two groups.

	CF (*n* = 26)	HC (*n* = 26)	*t*/χ^2^	*P*
Sex (male/female, *n*)*	13/13	13/13	0	1.000
Age (years)^#^	65.42 ± 5.15	65.38 ± 4.7	0.028	0.978
Edu (years)^#^	9.77 ± 3.98	10.96 ± 3.24	–1.184	0.242
BDI (scores)^#^	3.96 ± 1.93	4.62 ± 1.13	–1.49	0.144
MoCA (scores)^#^	19.31 ± 3.06	26.77 ± 0.86	–11.98	< 0.001
MQ (scores)^#^	77.54 ± 13.64	105.58 ± 5.32	–9.767	< 0.001
Average thickness of whole brain (mm)^#^	2.61 ± 0.06	2.63 ± 0.11	–1.208	0.233
Proportion of brain parenchyma^#^	0.73 ± 0.022	0.75 ± 0.033	–3.273	0.002
Total white fiber number^#^	30982.19 ± 6706.00	45014.62 ± 4539.86	–8.835	< 0.001
FI (scores)^#^	5.54 ± 0.76	2.19 ± 1.266	8.263	< 0.001
Grip strength (kg)^#^	23.03 ± 3.55	25.46 ± 3.83	–2.380	0.021
TIV (cm^3^)^#^	1332.19 ± 114.20	1386.00 ± 121.58	–1.645	0.106

Edu, education year; BDI, Beck Depression Inventory; MoCA, Montreal Cognitive Assessment; FI, frailty index; TIV, total intracranial volume; MQ, Wechsler’s Memory quotient; CF, cognitive frailty; HC, healthy control. *Chi-square test was used for data analysis. ^#^t-test was used for data analysis. Value of volume was presented by mean ± standard deviation. p***−****values for cognitive frailty vs. healthy control group comparison.*

### Hippocampal Subfield Volumes

Each side of the hippocampus was divided into 12 subregions; therefore, a total of 24 bilateral subregions were analyzed (see Figure 1). Six hippocampal subregions had significant differences in volume between the cognitive frailty and healthy control groups, including 4 from the left hippocampus and 2 from the right. Specifically, the volumes of the left presubiculum, parasubiculum, molecular layer of the HP, and HATA and the right CA1 and presubiculum in the cognitive frailty group were significantly lower than those in the healthy control group (*t* = −3.274, *p* = 0.002; *t* = −3.207, *p* = 0.002; *t* = −3.146, *p* = 0.003; *t* = −3.214, *p* = 0.002; *t* = −3.411, *p* = 0.001; *t* = −3.440, *p* = 0.001). No significant difference was found in the volumes of the other hippocampal subregions (see Table 2).

**TABLE 2:** Group comparison of hippocampal subregion volume.

	CF (*n* = 26)	HC (*n* = 26)	*t*	*p*
Left hippocampal tail	473.35 ± 73.38	495.29 ± 68.29	–1.116	0.270
Left subiculum	413.12 ± 50.07	427.54 ± 44.68	–1.096	0.278
Left CA1	585.32 ± 58.60	607.37 ± 56.26	–1.384	0.172
Left hippocampal fissure	156.39 ± 25.72	141.62 ± 15.83	2.492	0.016
Left presubiculum	275.91 ± 31.37	304.63 ± 31.88	–3.274	**0.002**
Left parasubiculum	56.80 ± 9.77	65.80 ± 10.45	–3.207	**0.002**
Left molecular layer HP	509.12 ± 39.06	545.08 ± 43.24	–3.146	**0.003**
Left GC-ML-DG	272.82 ± 33.77	277.24 ± 32.63	–0.479	0.634
Left CA3	188.22 ± 26.46	186.76 ± 29.98	0.186	0.853
Left CA4	238.12 ± 27.10	239.42 ± 27.56	–0.173	0.864
Left fimbria	74.02 ± 19.19	79.21 ± 21.65	–0.914	0.365
Left HATA	49.11 ± 6.37	56.02 ± 8.92	–3.214	**0.002**
Right hippocampal tail	514.38 ± 84.94	530.83 ± 72.21	–0.753	0.455
Right subiculum	419.32 ± 41.82	424.70 ± 39.21	–0.478	0.635
Right CA1	583.57 ± 45.85	634.11 ± 60.05	–3.411	**0.001**
Right hippocampal fissure	166.51 ± 26.14	162.41 ± 31.52	0.510	0.612
Right presubiculum	260.61 ± 29.01	284.17 ± 19.45	–3.440	**0.001**
Right parasubiculum	53.71 ± 11.97	56.20 ± 10.06	–0.812	0.421
Right molecular layer HP	547.02 ± 56.77	547.42 ± 64.26	–0.024	0.981
Right GC-ML-DG	290.37 ± 33.88	304.16 ± 28.16	–1.597	0.117
Right CA3	210.90 ± 28.38	207.79 ± 42.39	0.311	0.757
Right CA4	253.24 ± 27.55	249.10 ± 34.62	0.477	0.635
Right fimbria	68.48 ± 18.94	70.26 ± 18.07	–0.346	0.731
Right HATA	52.73 ± 6.69	53.89 ± 9.30	–0.519	0.606

**CF, cognitive frailty; HC, healthy control. Value of volume was presented by mean ± standard deviation, the unit of volume is cubic millimeter. Age, sex, education, Beck Depression Inventory, and TIV was considered as covariates. The value of *p* < 0.05 was expressed as bold values.**

### Correlation Between MoCA, MQ, FI Scores, and Hippocampal Subfield Volumes

**FIGURE 2:**
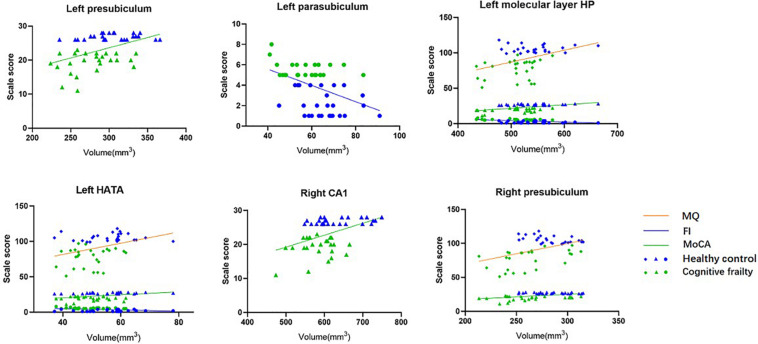
Correlation between hippocampal subfields volume and MQ, MoCA, frailty index scores (*n* = 52). After adjusting for age, sex, education years, BDI, and TIV.

After adjusting for age, sex, years of education, Beck Depression Inventory score and TIV, the volumes of the left molecular layer of the HP and HATA and the right presubiculum were positively correlated with the MQ score (*r* = 0.302, *p* = 0.039; *r* = 0.294, *p* = 0.044; *r* = 0.382, *p* = 0.008); the volumes of the left presubiculum, molecular layer of the HP, and HATA and the right CA1 and presubiculum were positively correlated with the MoCA scores (*r* = 0.302, *p* = 0.039; *r* = 0.374, *p* = 0.010; *r* = 0.258, *p* = 0.080; *r* = 0.321, *p* = 0.028; *r* = 0.325, *p* = 0.026); and the volumes of the left parasubiculum, molecular layer of the HP and HATA were negatively correlated with the frailty index score (*r* = −0.351, *p* = 0.016; *r* = −0.352, *p* = 0.015; *r* = 0.304, *p* = 0.038, see Table 3 and Figure 2).

**TABLE 3:** Partial correlation between hippocampal subregions volume and MQ, MoCA, frailty index scores (*n* = 52).

	MQ		MoCA		Frailty index	
	*r*	*p*	*r*	*p*	*r*	*p*
Left presubiculum	0.269	0.067	0.302	**0.039**	−0.270	0.066
Left parasubiculum	0.215	0.147	0.169	0.256	−0.351	**0.016**
Left molecular layer HP	0.302	**0.039**	0.374	**0.010**	−0.352	**0.015**
Left HATA	0.294	**0.044**	0.258	**0.080**	−0.304	**0.038**
Right CA1	0.241	0.103	0.321	**0.028**	−0.277	0.059
Right presubiculum	0.382	**0.008**	0.325	**0.026**	−0.286	0.052

**MQ, Wechsler’s Memory Quotient; MoCA, Montreal Cognitive Assessment; FI, Frailty index. Adjusting for age, sex, education years, Beck Depression Inventory score, and TIV. The value of *p* < 0.05 was expressed as bold values.**

## Discussion

Cognitive frailty is a simultaneous state of physical weakness and cognitive dysfunction. The pathogenesis of cognitive frailty is related to changes in brain structure. Our previous study found that the volumes of certain subcortical nuclei in cognitive frailty was smaller than those in healthy controls, indicating that the brain structure in cognitive frailty has indeed changed (Wan et al., 2020a). The current study showed that there was an obvious decrease in the volume of 6 hippocampal subregions, including the left presubiculum, parasubiculum, molecular layer of the HP, and HATA and the right CA1 and presubiculum. Furthermore, the volumes of the left molecular layer of the HP and HATA and the right presubiculum were positively correlated with MQ score, the volumes of the left presubiculum, molecular layer of the HP, and HATA and the right CA1 and presubiculum were positively correlated with the MoCA score, and the volumes of the left parasubiculum, molecular layer of the HP and HATA were negatively correlated with the frailty index. We also found that the cognitive frailty group showed a decreased proportion of brain parenchyma and total number of white matter fibers. These findings suggest that atrophy in some hippocampal subregions may be a potential mechanism underlying cognitive frailty.

To our knowledge, this is the first study to compare changes in hippocampal subfield volumes between cognitive frailty and healthy control. Anatomically, atrophied structures, including the presubiculum and parasubiculum, were situated at the medial portions of the hippocampus, while the molecular layer of the HP, CA1 and HATA were situated at the lateral portions. In fact, the volume of the presubiculum or parasubiculum, which is related to cognitive level, has been found to be decreased in many diseases, such as Parkinson’s disease, diabetes and Alzheimer’s disease (Low et al., 2019; Zhao et al., 2019; Li et al., 2020). The presubiculum and parasubiculum play an important role in cognitive processing and visual spatial function (Low et al., 2019). The volume of the presubiculum is considered to be a promising marker of imminent memory in Alzheimer’s disease (Jacobs et al., 2020). Additionally, the left presubiculum volume is positively correlated with MoCA score in MCI patients, which is in line with our study (Liang et al., 2018). The connection between the presubiculum and the retrosplenial cortex is the primary site of lesions in most forms of amnesia, and stimulation in this region is reported to enhance memory (Ferguson et al., 2019). Information transfer from the parasubiculum and presubiculum to the medial entorhinal cortex is key to controlling spatial navigation, an important cognitive function (Canto et al., 2019). Due to atrophy of the presubiculum and parasubiculum damaging these pathways, cognitive function might be weakened. Although we were unable to find any research on the relationship between the decline in physical function and the subiculum, some studies claimed that exercise could improve the functional connectivity or structural brain health of the parahippocampal gyrus and dentate gyrus of the hippocampus with areas related to motor, sensory integration and mood regulation (Tozzi et al., 2016; Varma et al., 2016). The parasubiculum might affect physical function through the above brain areas in cognitive frailty.

The hippocampal CA1 region is an important part of the medial temporal lobe memory circuit. It is selectively vulnerable to attack in the process of cognitive impairment, which can also predict episodic memory impairment (Ginsberg et al., 2019; La et al., 2019). Anatomical and physiological studies confirm that CA1 regulates hippocampal circuitry function and cognitive behavior by interacting with the entorhinal cortex, CA3, subiculum, and dentate gyrus (Xu et al., 2016). The results of animal experiments also showed that improvement in neuronal inactivation and apoptosis in the hippocampal CA1 area was significantly positively correlated with an improvement in cognitive function (Hei et al., 2019). The CA1 region has been highlighted in most studies as a focal atrophy subfield in the early stages of AD (de Flores et al., 2015). Our study shows that compared with that of healthy controls, the CA1 region of cognitive frailty patients was smaller and related to cognitive dysfunction, which was consistent with previous studies (Ogawa et al., 2019). As mentioned above, the CA1 region is an important node of information input and output. The results of this study showed that atrophy of the CA1 region in the cognitive frailty group was closely related to lower MoCA scores, which may be the secondary result of a decline in information processing ability stemming from this atrophy.

The HATA is located in the medial region of the hippocampus and is the transitional area between the hippocampus and amygdala. A study suggested that in Parkinson’s disease participants with cognitive impairment, the volume of the left fimbria, right CA1, and right HATA were decreased compared with those in cognitively healthy participants, the volumes of the left parasubiculum and HATA were predictive of the conversion from normal cognition to mild cognitive impairment, and the CA1 area was associated with baseline attention (Foo et al., 2017). The atrophy of the parasubiculum and HATA might destroy the integrity of the hippocampal-amygdala network, which is in charge of information processing (Zheng et al., 2018). In research on memory recall across the adult lifespan, it has been proposed that HATA is closely related to memory function, which is consistent with our study (Zheng et al., 2018). In addition, the HATA plays an important role in fear regulation, the underlying mechanism of situational learning and emotional memory (Fudge et al., 2012). Atrophy of the HATA might be related to a decline in the adaptability of elderly cognitive frailty adults to new environments, but further research is needed to confirm this hypothesis.

The molecular layer of the HP is located above the subiculum and underneath the fissure, which includes part of the subiculum and CA fields. A study on the development of hippocampal subregion volumes across adolescence found that CA1 and molecular layer of the HP were non-linear developmental trajectories in early volume increases, and global cognitive ability was positively associated with molecular layer of the HP development (Tamnes et al., 2018), while the numbers of synapses in the molecular layer of the HP showed a significant correlation with cognitive ability in participants with early Alzheimer’s disease, mild cognitive impairment, or no cognitive impairment (Scheff et al., 2006). Additionally, the loss of synapses might be an early event in the disease process, and this structural loss was correlated with cognitive function (Scheff et al., 2006). The current study found for the first time that the volume of the molecular layer of the HP was not only related to cognitive function but also negatively correlated with the frailty index. The reason for the negative correlation between the molecular layer of the HP and physical frailty is not clear and needs further study.

In terms of global brain parameters, we found that compared with that in the healthy control group, brain parenchyma atrophy was more severe and the total number of white matter fibers was smaller in the cognitive frailty group. These findings indicate that the degree of aging and complexity of the brain in elderly cognitive frailty patients are decreased from the overall level. In our study, the left side of the hippocampus lost more volume than the right side, which was different from previous work on Alzheimer’s disease (Parker et al., 2019). This might mean that physical frailty may be more associated with changes in the left side of the hippocampus.

### Limitations

In the present study, we focused on investigating alterations in hippocampal subfields, and the interpretation was largely limited to hippocampal function. The limited sample size of our research may have a certain impact on the accuracy of the results. A future study with a larger sample size is needed to replicate our findings. Second, due to the cross-sectional design of the study, the dynamic process of cognitive frailty could not be observed in real-time; therefore, the causality of the relationship was not clear. Since cross-sectional studies provide a limited ability to investigate relationships, a longitudinal design should be attempted in the future to gain deeper insights. Even so, the current study provides new information about the pathological progression of cognitive frailty.

## Conclusion

Our study shows that hippocampal subfield atrophy is more associated with the degree of cognitive impairment and physical frailty in the brains of older adults with cognitive frailty than in those of healthy controls. These findings indicate that specific hippocampal subfield volume changes might be involved in the pathological progression of cognitive frailty.

## Data Availability Statement

The raw data supporting the conclusions of this article will be made available by the authors, without undue reservation, to any qualified researcher.

## Ethics Statement

The studies involving human participants were reviewed and approved by the ethics committee of The Second People’s Hospital Affiliated with Fujian University of Traditional Chinese Medicine. The patients/participants provided their written informed consent to participate in this study. Written informed consent was obtained from the individual(s) for the publication of any potentially identifiable images or data included in this article.

## Author Contributions

GZ and LC designed the study. JT was responsible for coordinating and monitoring the process. MW and YY wrote the manuscript. HL, YX, SL, RX, JH, PQ, and CH managed the recruitment and data analysis. All the authors read and approved the final manuscript.

## Conflict of Interest

The authors declare that the research was conducted in the absence of any commercial or financial relationships that could be construed as a potential conflict of interest.

## Footnotes

1 http://www.freesurfer.net/

2 http://dbm.neuro.uni-jena.de/cat/

3 https://www.fil.ion.ucl.ac.uk/spm/

4 https://www.mathworks.com/products/matlab.html

5 https://fsl.fmrib.ox.ac.uk/fsl/fslwiki/Fslutils
